# The association of prior hospitalization with clinical outcomes among patients admitted with pneumonia: a propensity score matching study

**DOI:** 10.1186/s12879-019-3961-z

**Published:** 2019-04-27

**Authors:** Jae-Uk Song, Yee Hyung Kim, Mi Yeon Lee, Jonghoo Lee

**Affiliations:** 1 Division of Pulmonary and Critical Care Medicine, Department of Internal Medicine, Kangbuk Samsung Hospital, Sungkyunkwan University School of Medicine, Seoul, South Korea; 2 Department of Pulmonary and Critical Care Medicine, Kyung Hee University Hospital at Gangdong, School of Medicine, Kyung Hee University, Seoul, South Korea; 3 Division of Biostatistics, Department of R&D Management, Kangbuk Samsung Hospital, Sungkyunkwan University School of Medicine, Seoul, South Korea; 4 Department of Internal Medicine, Jeju National University Hospital, Jeju National University School of Medicine, Aran 13 gil 15, Jeju-si, Jeju, Special Self-Governing Province 690-767 South Korea

**Keywords:** Pneumonia, Hospitalization, Microbiology, Mortality, Antibiotics

## Abstract

**Background:**

Although prior hospitalization (PH) has been considered as a risk factor for infection with potentially drug-resistant (PDR) pathogens in patients admitted with pneumonia, the evidence is limited. We aimed to elucidate the clinical impact of PH on these patients.

**Methods:**

PH was defined as hospitalization for two or more days in the preceding 90 days. Patients with PH-associated pneumonia (PHAP) or community-acquired pneumonia (CAP) were matched using the propensity score matching method, and the clinical outcomes were compared. We also conducted subgroup analyses based on intravenous antibiotic use during PH, duration of PH, and time to re-admission.

**Results:**

A total of 704 patients were identified; the PHAP group included 97 patients (13.7%). After matching according to propensity scores, the baseline characteristics of the PHAP group were similar to those of the CAP group. The isolation rate of PDR pathogens as well as the 30-day and total in-hospital mortality did not differ between propensity score-matched PHAP and CAP patients (13.6% vs. 10.2%, *P* = 0.485; 10.2% vs. 14.8%, *P* = 0.362; and 13.6% vs. 15.9%, *P* = 0.671, respectively). In subgroup analyses, only intravenous antibiotic use during PH was associated with the isolation rate of PDR pathogens (adjusted OR: 5.066; 95% CI: 1.231–20.845).

**Conclusions:**

PH itself might not be related with higher isolation rates of PDR pathogens or mortality in patients admitted with pneumonia. Therefore, it seems reasonable that broad spectrum antibiotic therapy for PDR pathogens should be selectively applied to PHAP patients with intravenous antibiotic use during PH.

## Background

Pneumonia is a common cause of morbidity as well as the most common infectious cause of death in the world [1]. Based on microbiologic cultures and the clinical response of the patients, selecting appropriate antibiotics in patients with pneumonia is important [2]. To build management strategies for pneumonia, accurate assessment and classification of pneumonia are crucial.

It has been known that recent hospitalization prior to the occurrence of pneumonia was associated with potentially drug-resistant (PDR) pathogens and negatively influenced outcomes [2]. In the last decade, patients admitted with pneumonia who have had prior hospitalization (PH) for two or more days within the past 90 days have been represented as a subgroup of health care-associated pneumonia (HCAP), which is included in the spectrum of hospital-acquired pneumonia (HAP) and ventilator-associated pneumonia (VAP) [2]. However, there has been continuous controversy about the concept of HCAP because of excessive heterogeneity of HCAP definition and the inappropriateness in predicting the risk of PDR pathogens with subsequent excessive use of broad-spectrum antibiotics [3]. Consequently, the concept of HCAP was removed in the updated 2016 American Thoracic Society (ATS)/Infectious Diseases Society of America (IDSA) guidelines for the management of HAP and VAP [4]. However, individual analyses of particular HCAP populations or subgroups are still needed regarding risk factors for PDR pathogens and adverse outcomes.

There is little available information focusing on the clinical impact of PH in patients admitted with pneumonia. Instead, clinical characteristics of PH-associated pneumonia (PHAP) may be suggested within the findings from several HCAP studies [5–9]. According to these studies, PHAP accounts for 34.6 to 72% of HCAP, and patients with PHAP are at greater risk for colonization and infection with PDR pathogens [5–9]. Therefore, it has been suggested that they should be treated in a similar way to patients with late-onset HAP or VAP [2]. Also, previous studies have demonstrated that PH was a predictive factor of PDR pathogens in patients admitted with pneumonia [10–12]. But, because of limited information, it remained uncertain whether patients with PHAP should be treated empirically with antibiotics directed against PDR pathogens or not.

The aim of our study was to compare the clinical characteristics and the clinical outcomes of patients with PHAP versus community-acquired pneumonia (CAP) using propensity score matching analysis. Also, we conducted additional analyses to identify the determinant factors for the isolation of PDR pathogens and mortality in PHAP patients.

## Methods

### Study design

This retrospective observational cohort study was performed at Jeju National University Hospital (a 600-bed, university-affiliated hospital in Jeju, South Korea). Adult patients (≥ 18 years) who were hospitalized with pneumonia between January 2012 and December 2014 were investigated. Patients were screened by the Korean Standard Classification of Diseases-7 codes of the followings; J18.0–18.9 as representative codes of pneumonia in the primary discharge diagnosis [13]. We reviewed the medical records and radiological findings to confirm the diagnosis of pneumonia. According to the incidence of hospitalization for more than two days prior to the hospital visit of interest, we classified the study patients into PHAP and CAP groups. Demographics, radiological findings, laboratory findings, microbiological results, and clinical outcomes were compared between the two groups. We also conducted additional analyses to investigate the association between the rates of PDR pathogens/mortality in the PHAP subgroup and intravenous antibiotic use during PH, duration of PH > 10 days, and re-admission with pneumonia in ≤30 days. The study protocol was approved by the Ethical Review Committee of Jeju National University Hospital (IRB number 2017–04-005). Informed consent was waived because of the retrospective nature of the study.

### Definitions

PH was defined as hospitalization for two or more days in the preceding 90 days [2]. Pneumonia was defined according to previous studies [9, 14]. We excluded the following types of patients: (1) those who were readmitted due to pneumonia within 10 days of leaving the hospital (*n* = 13), (2) those who were transferred from other hospitals after hospitalization for > 48 h (*n* = 63), (3) those with obstructive pneumonia (*n* = 32), (4) those who had immunocompromised status (*n* = 21), such as those with neutropenia (absolute neutrophil count < 1500 cells/μL) after chemotherapy or human immunodeficiency virus infection, and (5) those who did not receive initial antibiotic treatment (*n* = 3).

HCAP was defined as a diagnosis of pneumonia in patients with any of the following: 1) recent history of hospitalization in an acute care hospital for ≥2 days in the past 90 days (PHAP), 2) residence in a nursing home or long-term care facility, 3) recent outpatient intravenous therapy or wound care within the past 30 days, or 4) attendance at a hospital clinic or dialysis center in the last 30 days [2]. CAP was defined as a diagnosis of pneumonia in patients who did not meet any of the criteria for HCAP. Patients with HAP were not included in the current analysis.

Changes in antibiotic regimens were defined as either escalation or de-escalation after culture sensitivities or clinical stabilities were identified. Inappropriate antibiotic therapy was noted if the empirical antibiotic treatment was not effective against the identified pathogen based on in vitro susceptibility testing [15]. Initial treatment failure was defined as death during initial treatment or a change of initial therapeutic agent after 48 h due to clinical instabilities [16].

### Microbiology and antibiotics

Sampling to determine the microbial etiology of pneumonia included sputum, tracheobronchial aspirates, bronchoalveolar lavage fluid, pleural fluid, or blood through a semiquantitative manner. The antibiotic sensitivity of all isolates was determined using a disc diffusion method. Serologic tests were performed to detect antibodies against *Mycoplasma pneumoniae* or *Chlamydia pneumoniae*. According to the clinical judgment of the attending physician, urinary antigen test for *Streptococcus pneumoniae* or *Legionella pneumophila* serogroup 1 was performed. Based on previous guidelines, methicillin-resistant *Staphylococcus aureus*, *Pseudomonas aeruginosa*, extended-spectrum beta-lactamase (ESBL)-producing or carbapenem-resistant *Klebsiella pneumoniae* and *Escherichia coli, Acinetobacter baumanii*, and *Stenotrophomonas maltophilia* were considered as PDR pathogens [2]. Atypical pathogens including *Mycoplasma pneumoniae*, *Chlamydophila* species, and *Legionella* species were considered to be fully susceptible to antibiotic therapy with macrolides and fluoroquinolones [17]. Based on the updated guidelines, patients who received a beta-lactam and/or quinolone were placed into the ‘CAP therapy’ group and those who received coverage for PDR pathogens were placed into the ‘HAP therapy’ group [4].

### Statistical analyses

The data are presented as median (interquartile range) for continuous variables and as number (%) for categorical variables. Continuous variables were compared using Student’s *t*-test for normally distributed variables and the Mann-Whitney *U* test for non-normally distributed variables. Categorical variables were compared using the Pearson χ^2^ test, and Fisher’s exact test was used when any cell contained less than 5 items.

To improve the balance of baseline characteristics and reduce the effect of selection bias and potential confounding in this retrospective cohort study, estimated propensity scores were used to match the patients with PHAP to those with CAP using logistic regression. All variables were included in the propensity models except for the outcome variables: age, sex, aspiration tendency, tube feeding, malignancy, chronic liver disease, chronic heart disease, chronic kidney disease, diabetes mellitus, chronic respiratory disease, central nervous system disorder, two or more comorbidities, body temperature, altered mental state, respiratory failure, sepsis or septic shock at onset, intensive care unit admission, need for ventilation, multi-lobar involvement, pleural effusion, antibiotics regimens, white blood cells, C-reactive protein, and PSI score.

In our study, the model was computed for each of the patients using a logistic regression model and then used to match individuals in the PHAP group to individuals in the CAP group using 1:1 nearest neighbor matching [18]. To ensure balanced matches, a caliper, maximum allowable difference between two groups, was defined as 0.2 (i.e., 0.2 X standard deviation of the logit of the propensity scores) resulting in a relatively narrow difference between matched subjects [18]. Standardized mean differences were estimated for all baseline covariates before and after matching to assess pre-match imbalance and post-match balance. Logistic regression analyses were also conducted to assess the association between PHAP and clinical outcomes before and after adjusting for propensity scores.

In addition, we performed multivariable logistic regression analysis in the subgroup of patients with PHAP to identify factors associated with secondary outcomes, as measured by the estimated odds ratio (OR) with 95% confidence intervals (CI). All tests were two-sided, and *P*-values < 0.05 were considered statistically significant. All statistical analyses were performed with SPSS software package, version 24 (SPSS Inc., Chicago, IL, USA).

## Results

### Baseline characteristics of the PHAP and CAP groups

**Fig. 1:**
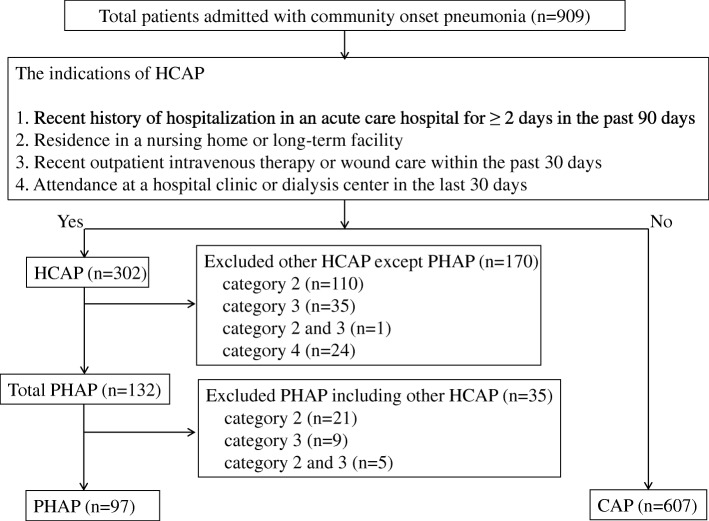
Flow diagram of patient enrollment. CAP: Community-acquired pneumonia, HCAP: healthcare-associated pneumonia, PHAP: Prior hospitalization-associated pneumonia

Figure 1 shows a flow diagram for the identification of the study populations. Among 909 patients identified, 97 (10.6%) had PHAP and 607 (66.7%) had CAP. Since the remaining 205 patients (22.5%) were diagnosed as having one or more category of HCAP other than PHAP, they were ultimately excluded from the present study. Among PHAP patients, 6 were admitted to the ICU during the PH period and 3 underwent mechanical ventilation. The baseline characteristics of the patients with PHAP and CAP are presented in Table I. Patients with PHAP had more comorbidities than those with CAP. In terms of clinical parameters, respiratory failure, sepsis or septic shock at onset, and the admission rate to the ICU were more prevalent in patients with PHAP. And PHAP patients received more frequently initial HAP therapy than overall CAP patients. In addition, the median PSI scores were higher in patients with PHAP than in those with CAP.

The propensity score matching process was performed according to multiple logistic regression model. From the model, a fitted probability (propensity score) for each subject was calculated for how likely they are to be in the PHAP group based on their covariate profile of matching variable values. These 704 propensity scores were divided into four groups, from top to bottom: unmatched PHAP (*n* = 11), matched PHAP (*n* = 88), matched CAP (*n* = 88), unmatched CAP (*n* = 519). After the propensity score matching process, the distributions of the different baseline co-morbidities, clinical parameters, initial HAP therapy and severity indexes were well balanced between the PHAP and propensity score-matched CAP groups (Table 1). The average standardized mean difference was 0.336 before matching, and decreased to 0.085 after matching.

**Table 1:** Baseline characteristics of the patients

Characteristics	Overall series				Propensity score-matched pairs			
	PHAP (*n* = 97)	CAP (*n* = 607)	*P* value	D	PHAP (*n* = 88)	CAP (*n* = 88)	*P* value	D
Age, years	72 (65–78)	71 (58–79)	0.315	0.022	72 (65–69)	72 (64–81)	0.666	0.038
Male	64 (65.9)	358 (58.9)	0.191	0.165	55 (62.5)	59 (67.0)	0.528	0.109
Aspiration tendency^a^	22 (22.6)	106 (17.4)	0.216	0.180	20 (22.7)	21 (23.9)	0.858	0.035
Tube feeding	4 (4.1)	8 (1.3)	0.070	0.675	4 (4.5)	5 (5.7)	1.000	0.129
Comorbidity								
Malignancy^b^	36 (37.1)	90 (14.8)	< 0.001	0.673	27 (30.7)	31 (35.2)	0.521	0.113
Chronic liver disease^c^	5 (5.1)	36 (5.9)	0.762	0.082	4 (4.5)	4 (4.5)	1.000	0
Chronic heart disease^d^	26 (26.8)	79 (13.0)	< 0.001	0.494	22 (25.0)	17 (19.3)	0.364	0.182
Chronic kidney disease^e^	7 (7.2)	64 (10.5)	0.312	0.229	7 (8.0)	9 (10.2)	0.600	0.152
Diabetes mellitus^f^	31 (31.9)	125 (20.5)	0.012	0.328	27 (30.7)	30 (34.1)	0.629	0.085
Chronic respiratory disease^g^	42 (43.2)	153 (25.2)	< 0.001	0.451	38 (43.2)	33 (37.5)	0.442	0.130
Central nervous system disorders^h^	19 (19.5)	100 (16.4)	0.447	0.116	19 (21.6)	20 (22.7)	0.856	0.036
Two or more comorbidities	45 (46.3)	193 (31.7)	0.005	0.341	40 (45.5)	36 (40.9)	0.543	0.102
Clinical parameters								
Body temperature (°C)	37.5 (36.8–38.2)	37.5 (36.9–38.3)	0.943	0.044	37.5 (36.8–38.2)	37.7 (37.1–38.3)	0.442	0.054
Altered mental state^i^	10 (10.3)	36 (5.9)	0.105	0.331	8 (18.1)	7 (8.0)	0.787	0.080
Respiratory failure^j^	52 (53.6)	161 (26.5)	< 0.001	0.642	43 (48.9)	42 (47.7)	0.880	0.025
Sepsis or septic shock at onset^k^	21 (21.6)	63 (10.3)	0.001	0.479	16 (18.2)	18 (20.5)	0.703	0.080
Intensive care unit admission	19 (19.5)	66 (10.8)	0.014	0.381	16 (18.2)	13 (14.8)	0.542	0.137
Need for ventilation	5 (5.1)	43 (7.0)	0.484	0.119	5 (5.7)	4 (4.5)	1.000	0.129
Radiological findings								
Multi-lobar involvement	43 (44.3)	271 (44.6)	0.954	0.007	37 (42.0)	36 (40.9)	0.878	0.025
Pleural effusion	18 (18.5)	115 (18.9)	0.928	0.014	16 (18.2)	14 (15.9)	0.688	0.088
Initial antibiotics								
CAP targeted therapy	57 (58.8)	526 (86.7)	< 0.001	0.836	57 (64.8)	61 (69.3)	0.521	0.113
HAP targeted therapy	40 (41.2)	81 (13.3)	< 0.001	0.836	31 (35.2)	27 (30.7)	0.521	0.113
Laboratory findings								
White blood cells (/mm^3^)	11,400 (7100-15,850)	10,600 (7900-14,500)	0.712	0.116	12,100 (7525-16,175)	12,400 (9225-15,850)	0.270	0.017
C-reactive protein (mg/dl)	9.1 (4.1–17.0)	9.8 (4.0–17.0)	0.698	0.034	8.4 (3.9–16.1)	9.8 (4.0–17.1)	0.644	0.045
Indices for disease severity								
PSI score	120 (91–137)	89 (67–117)	< 0.001	0.647	113 (87–134)	114 (92–140)	0.492	0.129

Data are presented as median (interquartile range) or number (%)

CAP, community-acquired pneumonia; CURB-65, Confusion, Urea, Respiratory rate, Blood pressure, Age ≥ 65; D, standardized mean difference; HAP, hospital-acquired pneumonia; MRSA, methicillin-resistant *Staphylococcus aureus*; PHAP, prior hospitalization associated with pneumonia; PSI, Pneumonia Severity Index;

^a^Aspiration tendency was defined as having factors predisposing a patient to aspiration, such as a bed-ridden state, central nervous system or oropharyngeal disorders (e.g., malignancy), gastroesophageal disorders (e.g., esophageal diverticulum, achalasia, systemic sclerosis, esophageal cancer, severe reflux esophagitis, or post-gastrectomy), Levin tube inserted state, and subjective and/or observed aspiration/choking/dysphagia/vomiting episode

^b^Malignancy included active at the time of presentation or requiring anticancer treatment within the previous five years

^c^Chronic liver disease included pre-existing viral or toxic hepatopathy at the time of pneumonia diagnosis

^d^Chronic heart disease was identified based on past history or administration of diuretics for treatment of heart disease

^e^Chronic kidney disease included pre-existing renal disease with documented abnormal serum creatinine levels

^f^Diabetes mellitus included a history of diagnosis of intolerance to glucose, hemoglobin A1c ≥6.5% or treatment with oral hypoglycemic agents or insulin

^g^Chronic respiratory disease included simple chronic bronchitis, chronic obstructive pulmonary disease, and structural lung diseases such as bronchiectasis and interstitial lung diseases

^h^Central nervous system disorders included acute or chronic vascular or nonvascular encephalopathy with or without dementia

^i^Altered mental state was defined as Glasgow Coma Score ≤ 13 documented by the physician

^j^Respiratory failure was defined when PaO2 was 60 mmHg or less or when PaO2/FiO2 ratio was 300 mmHg or less

^k^Sepsis was defined as organ dysfunction identified as an acute change in the Sequential Organ Failure Assessment score ≥ 2 consequent to pneumonia. Septic shock was defined as sepsis with persisting hypotension requiring vasopressors to maintain mean arterial pressure ≥ 65 mmHg and having a serum lactate level > 2 mmol/L (18 mg/dL) despite adequate volume resuscitation

### Microbial etiology and initial antibiotic therapy

**Fig. 2:**
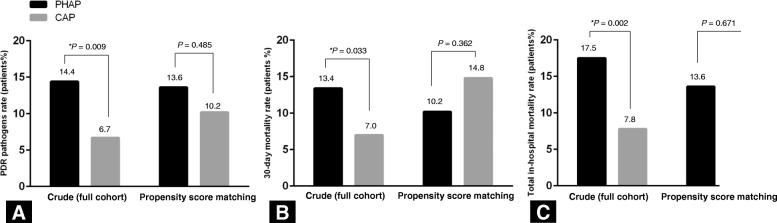
Associations between the type of pneumonia and clinical outcomes in (**a**) PDR pathogens rate, (**b**) 30-day mortality rate, and (**c**) total in-hospital mortality rate. ^*^P < 0.05 (statistical significance). PDR: Potentially drug-resistant

The distributions of microorganisms and initial antibiotics are shown in Table 2. Overall etiology was established in 33 (34.0%) and 201 (33.1%) patients in the PHAP and the CAP groups, respectively (Table 2). In both groups, the most frequently identified microorganism was *S. pneumoniae*. The rate of PDR pathogen identified was significantly higher in the PHAP patients (14.4% vs. 6.7%, *P* = 0.009, Fig. 2a). *P. aeruginosa* was the microorganism that was isolated the most frequently among PDR pathogens and was more common in the PHAP patients (8.2% vs. 3.1%, *P* = 0.023). However, following propensity score matching, there were no differences in the incidences of PDR pathogens between the two groups (13.6% vs. 10.2%, *P* = 0.485, Fig. 2a).

**Table 2:** The microorganisms isolated from the patients and initial antibiotics

Variables	Overall series			Propensity score-matched pairs		
	PHAP (*n* = 97)	CAP (*n* = 607)	*P* value	PHAP (*n* = 88)	CAP (*n* = 88)	*P* value
Microorganisms						
Identified pathogens	33 (34.0)	201 (33.1)	0.860	30 (34.1)	35 (39.8)	0.435
Gram-positive bacteria						
*Streptococcus pneumoniae*	9 (9.2)	85 (14.0)	0.193	8 (9.1)	10 (11.4)	0.619
*Staphylococcus aureus*	5 (5.1)	21 (3.4)	0.386	4 (4.5)	5 (5.7)	1.000
Methicillin-sensitive *S. aureus*	2 (2.0)	9 (1.4)	0.654	2 (2.3)	3 (3.4)	1.000
Methicillin-resistant *S. aureus*	3 (3.0)	12 (1.9)	0.448	2 (2.3)	2 (2.3)	1.000
Other gram-positive bacteria	1 (1.0)	8 (1.3)	1.000	1 (1.1)	0 (0)	1.000
Gram-negative bacteria						
*Pseudomonas aeruginosa*	8 (8.2)	19 (3.1)	0.023	8 (9.1)	7 (8.0)	0.787
*Haemophilus influenza*	1 (1.0)	7 (1.1)	1.000	1 (1.1)	1 (1.1)	1.000
*Klebsiella pneumoniae*	8 (8.2)	31 (5.1)	0.209	7 (8.0)	8 (9.1)	0.787
ESBL (+)	0 (0)	2 (0.3)	1.000	0 (0)	0 (0)	1.000
ESBL (−)	8 (8.2)	29 (4.7)	0.155	7 (8.0)	8 (9.1)	0.787
*Acinetobacter* species	2 (2.0)	2 (0.3)	0.094	2 (2.3)	0 (0)	0.497
Other gram-negative species^a^	1 (1.0)	11 (1.8)	1.000	1 (1.1)	1 (1.1)	1.000
*Mycoplasma pneumonia*	2 (2.0)	27 (4.4)	0.409	2 (2.3)	3 (3.4)	1.000
Polymicrobial pathogens	4 (4.1)	10 (1.6)	0.114	4 (4.5)	0 (0)	0.121
Potentially drug-resistant pathogens^b^	14 (14.4)	41 (6.7)	0.009	12 (13.6)	9 (10.2)	0.485

Data are presented as number (%). Percentages refer to dividing by the total number of patients

CAP, community-acquired pneumonia; ESBL, extended-spectrum β-lactamase; HAP, hospital-acquired pneumonia; PHAP, prior hospitalization associated with pneumonia

^a^Other gram-negative species included *Escherichia coli, Enterobacter species, Serratia marcescens,* and *Legionella pneumophilia*

^b^Multidrug resistant pathogens included methicillin-resistant *Staphylococcus aureus* (MRSA), *Pseudomonas* species, *Acinetobacter* species, *Stenotrophomonas maltophilia*, and ESBL-producing Enterobacteriaceae

### Clinical outcomes

The clinical outcomes of the patients with either PHAP or CAP are shown in Table 3. The median length of hospital stay was longer in patients with PHAP than in those with CAP (9 vs. 7 days, *P* = 0.001). The 30-day and total in-hospital mortality rates were also higher in the patients with PHAP (13.4% vs. 7.0%, *P* = 0.033, and 17.5% vs. 7.8%, *P* = 0.002, respectively, Fig. 2b and c). However, after propensity score matching, there were no significant differences in these variables between groups (Fig. 2b and c).

**Table 3:** Clinical outcomes of the patients

Clinical outcomes	Overall series			Propensity score-matched pairs		
	PHAP (*n* = 97)	CAP (*n* = 607)	*P* value	PHAP (*n* = 88)	CAP (*n* = 88)	*P* value
Duration of antibiotic therapy (days)	10 (7–12)	10 (7–13)	0.536	10 (7–12)	10 (7–14)	0.426
Change of antibiotics	17 (17.5)	122 (20.0)	0.554	16 (18.2)	25 (28.4)	0.109
Escalation	1 (1.0)	12 (2.0)	1.000	1 (1.1)	3 (3.4)	0.621
De-escalation	16 (16.5)	110 (18.1)	0.698	15 (17.0)	22 (25.0)	0.195
Use of inappropriate antibiotics^a^	4 (12.1)	39 (19.4)	0.317	4 (4.5)	9 (10.2)	0.150
Failure of initial antibiotic therapy	20 (20.6)	113 (18.6)	0.640	17 (19.3)	23 (26.1)	0.280
Length of hospital stay (days)	9 (6–14)	7 (5–11)	0.001	9 (6–13)	8 (6–15)	0.871
30-day mortality rate	13 (13.4)	43 (7.0)	0.033	9 (10.2)	13 (14.8)	0.362
Total in-hospital mortality rate	17 (17.5)	48 (7.8)	0.002	12 (13.6)	14 (15.9)	0.671

Data are presented as median (interquartile range) or number (%)

*CAP* community-acquired pneumonia, *PHAP* prior hospitalization associated with pneumonia

^a^The proportion was calculated by dividing the number of patients with identified pathogens

By logistic regression analysis, when compared to overall CAP patients, the presence of PHAP had a significant association with increased rate of PDR pathogens identified as well as 30-day and total in-hospital mortality rates (Table 4). However, in propensity score–matched patients, there were no significant differences for these outcomes between PHAP and CAP patients (Table 4).

**Table 4:** The associations between PHAP and clinical outcomes before and after propensity score matching

Variables	Odds ratio	95% confidence interval	*P* value
Potentially drug-resistant pathogens			
Crude (full cohort)	2.329	1.217–4.456	0.011
Propensity score matching	1.386	0.552–3.477	0.487
30-day mortality rate			
Crude (full cohort)	2.030	1.048–3.933	0.036
Propensity score matching	0.657	0.265–1.628	0.364
Total in-hospital mortality rate			
Crude (full cohort)	2.475	1.357–4.513	0.003
Propensity score matching	0.835	0.362–1.923	0.671

### Subgroup analysis in patients with PHAP

**Fig. 3:**
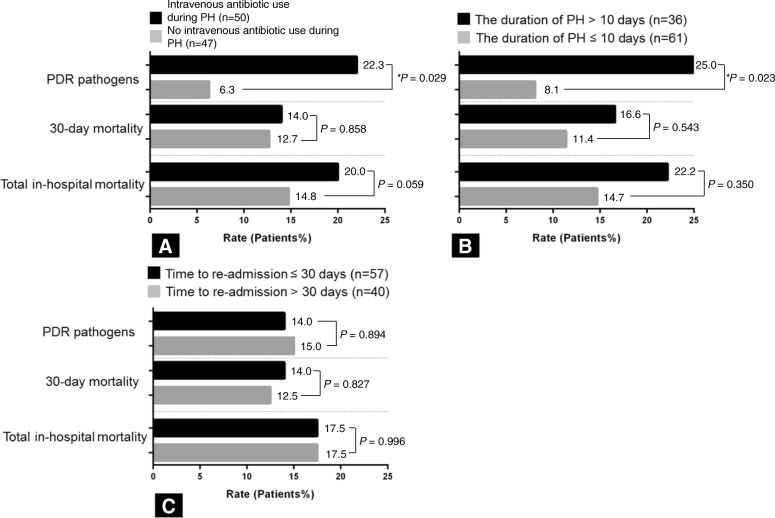
The isolation rate of PDR pathogens, 30-day mortality rate, and total in-hospital mortality rate according to (**a**) intravenous antibiotic use during PH, (**b**) duration of PH, and (**c**) time to re-admission in patients with prior hospitalization-associated pneumonia. ^*^P < 0.05 (statistical significance). PDR: Potentially drug-resistant; PH, Prior hospitalization

PHAP patients with intravenous antibiotic use during PH or a duration of PH > 10 days showed a significantly higher prevalence of PDR pathogens compared with those with no intravenous antibiotic use during PH or with PH ≤ 10 days (22.0% vs. 6.3%, *P* = 0.029, Fig. 3a and 25.0% vs. 8.1%, *P* = 0.023, Fig. 3b). There were no significant differences among the groups regarding the rates of 30-day and total in-hospital mortality (Fig. 3a and b). By multivariable analysis, intravenous antibiotic use during PH was independently associated with the isolation rate of PDR pathogens (OR: 5.066, 95% CI: 1.231–20.845, *P* = 0.025, Table 5). In patients with PHAP who received intravenous antibiotic during PH, the isolated PDR pathogens are *P. aeruginosa* (*n* = 5), MRSA (*n* = 3), ESBL producing *E. coli* (*n* = 2), *Acinetobacter baumanii* (*n* = 1), and ESBL producing *K. pneumoniae* (*n* = 1), allowing for overlap. In addition, when 57 PHAP patients re-admitted within 30 days were compared with 40 PHAP who were re-hospitalized after 30 days, the rate of PDR pathogens isolated and mortality did not differ significantly between the two groups (Fig. 3c).

**Table 5:** Multivariate logistic regression analysis for predictive factors associated with potentially drug-resistant pathogens in patients admitted with prior hospitalization-associated pneumonia

Risk factors	Odds ratio	95% Confidence interval	*P* value
Intravenous antibiotic use during prior hospitalization	5.066	1.231–20.845	0.025
Duration of prior hospitalization > 10 days	0.392	0.118–1.300	0.126
Time to re-admission ≤30 days	1.704	0.495–5.863	0.398

## Discussion

The present study demonstrated no significant differences in the isolation rates of PDR pathogens, 30-day mortality, or total in-hospital mortality between patients with PHAP and those with CAP after propensity score matching and competing risk adjustment. Also, in subgroup analysis, the significant risk factor for the isolation of PDR pathogens in patients with PHAP was intravenous antibiotic use during PH. Our results therefore suggest that PDR pathogen-targeted antibiotic therapy should be considered in selected patients, such as those received intravenous antibiotic use during PH, rather than in all patients with PHAP.

The concept of HCAP was eliminated in the revised 2016 ATS/IDSA guidelines for the management of HAP and VAP [4]. However, because interaction with the healthcare system is potentially a risk for PDR pathogens, the concept of HCAP as a separate clinical entity would be still reasonable [4]. Also, new ATS/IDSA guidelines stated that HCAP could be included in the upcoming CAP guidelines because patients with HCAP frequently presented from the community and were initially cared for in emergency departments [4]. Several studies revealed that HCAP and drug-resistant bacterial pneumonia may not share identical risk factors for PDR pathogens, and the broad-spectrum antibiotic treatment targeting PDR pathogens in patients with HCAP may not be adequate and can even lead to overtreatment [11].

PH has been considered as a risk factor for PDR pathogens in patients admitted with pneumonia [10–12]. In a previous prospective study, PH was an independent predictive factor of gram-negative bacteria and *P. aeruginosa* in patients with CAP [10]. In addition, a recent retrospective study demonstrated that PH was independently associated with PDR pathogens in immunocompetent patients admitted with pneumonia [11]. PH was also a risk factor for PDR pathogens in HCAP patients [12]. Compared to the non-PDR pathogens group, the PDR pathogens group had a higher prevalence rate of PH [12]. However, these studies had a critical weakness in that there were significant differences in the baseline characteristics between groups [10–12]. Before adjustment for baseline characteristics, the present study also had differences similar to those of previous studies in terms of baseline characteristics. Due to these differences in baseline characteristics, the isolation rate of PDR pathogens was higher in patients with PHAP. After the propensity score matching process, there was no significant difference in the isolation rate of PDR pathogens between the two groups. Therefore, it seems that more comorbidities and higher severity of pneumonia account for the increased isolation rate of PDR pathogens in patients admitted with PHAP. Our results were consistent with the findings of a prospective multicenter case–control study performed in Spain [19]. Therefore, our findings might lead to the need for a reassessment of the recommendation to consider the presence of PH for the selection of patients with PDR pathogens [2].

We also investigated the mortality rate of PHAP patients compared to those with CAP. Patients with PHAP had higher 30-day and total in-hospital mortality rates. These were reduced to nonsignificant associations after adjustment for baseline characteristics. The lack of association between PH and mortality would be reflected in previous HCAP studies. A recent meta-analysis reported that the excess mortality of HCAP patients seemed to be primarily related to underlying host-related factors rather than the presence of the category of HCAP such as PH [20]. Similar results were reported in a prospective observational study to determine the impact of HCAP on 30-day mortality, where PHAP patients accounted for 37.9% of total HCAP patients, and there was no relationship between HCAP and 30-day mortality (adjusted OR: 0.97, 95% CI: 0.61–1.55; *P =* 0.9) [8].

Although the presence of PH could serve to guide clinicians in identifying patients at high risk of PDR pathogens, its automatic application in all patients with PHAP might lead to overuse of PDR pathogen-targeted antibiotics for several reasons. First, PH is traditionally defined as a recent admission for two or more days within the past 90 days [2] and has been a crucial factor in previous HCAP studies. However, PH criteria differed among studies. The interval between PH and current pneumonia varied from 30 to 365 days [6, 8, 15, 21–26]. Second, by definition, PHAP cannot avoid including patients at low risk of PDR pathogens who are hospitalized due to a simple surgery or procedure and work-up of specific diseases. Namely, the current definition of PH is too broad to predict patients with PDR pathogens. Therefore, we performed subgroup analyses of three variables: intravenous antibiotic use during PH, duration of PH > 10 days, and re-admission with pneumonia in ≤30 days. To identify the predictive factor for PDR pathogens or mortality among these variables, we additionally performed multivariable logistic regression analysis. In the present study, it is notable that intravenous antibiotic use during PH was a significant risk factor that was strongly associated with PDR pathogens. Our findings are similar to the results reported by several previous studies [12, 23, 27]. This elicits the recommendation that we may consider the presence of intravenous antibiotic use during PH, rather than PH itself, when selecting antimicrobial regimens.

To the best of our knowledge, this is the first study to evaluate the clinical impact of PH in patients admitted with pneumonia and to identify predictive factors for PDR pathogens in patients with PHAP. In addition, the present study has the strength of the propensity score-matching process, although the patients were a retrospective cohort. Meanwhile, there are some study limitations. First, because our study was performed retrospectively at a single center and the number of PHAP patients was relatively small, the results should be carefully interpreted, and its findings may not be generalizable to other institutions. Second, the selection of treatment strategies including antibiotics was at the discretion of the physicians. And although we performed propensity score-matched analyses to adjust for these potential confounding factors, a reduction of the original population with subsequent loss of statistical power inevitably occurred. Third, we included patients with negative culture results on distribution of PDR pathogens. Because microorganisms were only identified in 34.0 and 33.1% of the PHAP and the CAP patients, respectively, this might not reflect the real isolation rate of PDR pathogens. Finally, in subgroup analysis, we revealed that intravenous antibiotic use during PH was associated with the isolation of PDR pathogens in patients with PHAP. However, since our study was retrospective in nature, we could not investigate details such as the classes of intravenous antibiotic use during PH or their duration and patients who only received oral antibiotics prior to admission. These can lead to potential bias in the present study.

## Conclusions

Patients with PHAP had more comorbidities and higher pneumonia severity than those with CAP. Because these patients had primarily worse host-related factors associated with pneumonia rather than PH itself, they seemed to have poorer clinical outcomes. The independent risk factor for the isolation of PDR pathogens was intravenous antibiotic use during PH in patients with PHAP. Our findings would be valuable in that key potential confounders were adjusted through the propensity score matching process. Therefore, we suggest that empirical antibiotics with nosocomial coverage might be considered for selected PHAP patients who received intravenous antibiotic use during PH.

## Abbreviations

ATS/IDSA: American thoracic society/infectious diseases society of America
CAP: Community-acquired pneumonia
CRP: C-reactive protein
CURB-65: Confusion, urea, respiratory rate, blood pressure, age ≥ 65
ESBL: Extended-spectrum β-lactamase
HAP: Hospital-acquired pneumonia
HCAP: Healthcare-associated pneumonia
ICU: Intensive care unit
IQRs: interquartile ranges
MRSA: Methicillin-resistant *Staphylococcus aureus*
PDR: potentially drug-resistant
PH: Prior hospitalization
PHAP: PH-associated pneumonia
PSI: Pneumonia severity index
VAP: Ventilator-associated pneumonia
WBC: White blood cell
